# Supplementary material for: Conditional chemoconnectomics (cCCTomics) as a strategy for efficient and conditional targeting of chemical transmission
Source: eLife. 2024 Apr 30;12:RP91927. doi: 10.7554/eLife.91927 (PMC11060718; doi:10.7554/eLife.91927)
Supplement: Figure 3—source data 3. [file elife-91927-fig3-data3.zip › Figure 3 source data3 part 1.zip!/Figure 3-Source data 3-part1/1081-1-CRISPResso_Report_8zosk6yj.zip!/CRISPResso_on_1081_3/2b.Nucleotide_percentage_quilt_around_sgRNA_tttctggactacagtcccca.pdf]

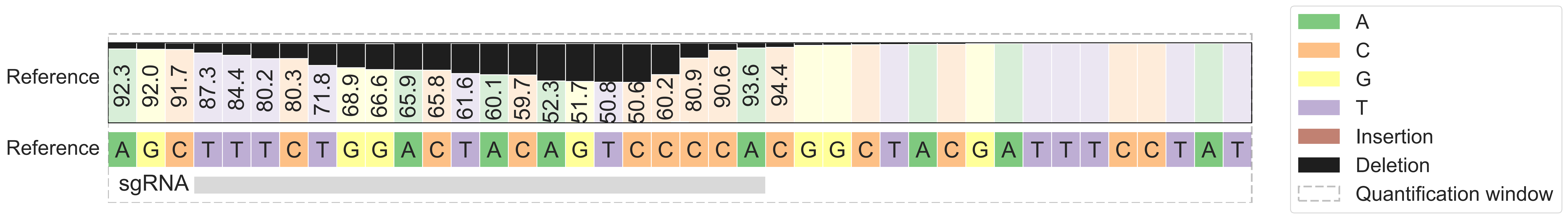
Reference
92.3
92.0
91.7
87.3
84.4
80.2
80.3
71.8
68.9
66.6
65.9
65.8
61.6
60.1
59.7
52.3
51.7
50.8
50.6
60.2
80.9
90.6
93.6
94.4
Reference
A G C T T T C T G G A C T A C A G T C C C C A C G G C T A C G A T T T C C T A T
sgRNA
A
C
G
T
Insertion
Deletion
Quantification window
